# Assessment of carotid artery ultrasonography in the presence of an acoustic shadow artifact

**DOI:** 10.1186/s12883-019-1405-4

**Published:** 2019-07-29

**Authors:** Estelle E. Seyman, Natan Bornstein, Eitan Auriel, Oren Cohen, Tania Nissel, Hen Hallevi

**Affiliations:** 1 Division of Neurology, Stroke Department, Tel-Aviv Sorasky Medical Center, 6 Weizmann St., Tel Aviv-Jaffa, Israel; 2 Department of Neurology Shaarey-Tzedek Medical Center, 12 Shmuel Bait Street, Jerusalem, Israel; 3 Department of Neurology, Rabin Medical Center, Petah Tikva, Israel; 4 Sackler School of Medicine, Tel Aviv University, Tel Aviv-Jaffa, Israel

**Keywords:** Ischemic stroke, Carotid artery stenosis, Acoustic shadow, Color Doppler ultrasound

## Abstract

**Background:**

B-mode and Color Doppler ultrasonography (CDUS) are the methods of choice for screening and determining the degree of Carotid artery stenosis. The evaluation of stenosis with calcification may be hampered by a common CDUS artifact known as acoustic shadow (AS). Our objective was to assess the change in reliability of CDUS readings in the presence of an AS artifact.

**Methods:**

Single center retrospective observational study. Included were patients with either an AS artifact or high-grade stenosis (defined by peak systolic velocity (PSV) > 240 cm/s) demonstrated in CDUS, and had a CT angiography (CTA) done within 6 months of the sonographic exam. All subjects were identified through the Tel-Aviv Sorasky medical center (TASMC) CDUS unit registry from which clinical information was extracted. CDUS images were manually reviewed grading AS magnitude. All CTAs were reviewed and reconstructed for accurate assessment of percent stenosis and were used as gold standard.

**Results:**

The study cohort included 227 consecutive patients (corresponding with 454 internal carotid arteries) meeting inclusion criteria. 43.2% of the arteries (*n* = 195) had an AS artifact present on CDUS, regardless of percent stenosis, with a large artifact present in 6.7% arteries (*n* = 30). Older age was significantly related to the presence of AS artifact (*p* < 0.001). In the study cohort as a whole there was a strong correlation between percent stenosis on CTA and PSV values (Pearson’s r 0.672, p < 0.001) regardless of AS existence. The CDUS sensitivity and specificity for predicting severe stenosis were 82 and 73% respectively. The presence of a small AS slightly diminished the correlation between CDUS and CTA results without compromising CDUS reliability. A large AS severely affected the correlation between CDUS and CTA exams (Pearson’s r = 0.24, *p* = 0.27) and reduced CDUS reliability with a sensitivity and specificity of 62%.

**Conclusion:**

The presence of a large AS severely degrades the accuracy of the routine CDUS measurements. In these cases, the patient should be referred to a CDUS exam including doppler-measurement of periorbital arteries and intracranial arteries in addition to other imaging modalities such as CTA or MRA in order to assess future stroke risk.

## Background

Carotid artery stenosis (CS) is a well-established risk factor for ischemic stroke [1] with degree of stenosis being one of the most important risk determinants. Management and treatment of CS either by Carotid endarterectomy or stenting is considered an effective means for stroke prevention especially in symptomatic cases [2]. As a result, much research has been dedicated to determining sensitive, specific, and cost-effective techniques to assess and follow CS [1].

B-mode and Color Doppler Ultrasound (CDUS) are currently the methods of choice for screening and determining the degree of CS [3]. CDUS relies on changes in the Internal Carotid artery flow velocity and waveform patterns throughout the cardiac cycle; ie, Peak systolic velocity (PSV) and End Diastolic velocity (EDV). PSV is the most reproducible reading and thus most commonly used [3, 4]. The threshold of PSV > 130 cm/s is associated with sensitivity of 98% (95% CI 97–100%) and specificity of 88% (95% CI, 76–100%) for the identification of angiographic CS of > 50% calculated according to the NASCET method. A PSV > 240 cm/s has a sensitivity of 90% (95% CI, 84 to 94%) and a specificity of 94% (95% CI, 88 to 97%) for the diagnosis of CS of > 70% calculated according to the NASCET method [3]. Additional measures deriving from PSV and EDV velocity readings are the Pulsatile Index (PI) and Resistivity Index (RI). The PI and RI are considered to be surrogate markers for arterial stiffness [5] and were shown to be related to intra cranial small vessel disease [5, 6]. These indices change with the ultrasound flow pattern, reflecting attenuated flow and low-resistance flow in post-stenotic areas and might add important information when CDUS artifacts are present.

CDUS suffers limitations such as apparatus-specific artifacts, lack of consistent inter- and intra-observer agreement, and poor signal-to-noise ratio [1, 7]. Furthermore, the evaluation of stenosis with calcification may be hampered by CDUS artifacts such as an acoustic shadow (AS) [8].

Another common and accurate imaging modality of the carotid artery is Computed Tomography Angiography (CTA) [9] . It has high reliability in assessing the exact extent of stenosis and images the vascular anatomy [1, 9–11]. None the less, CTA has its interpretation pitfalls and carries risks associated with the use of iodinated contrast agents, as well as radiation exposure [9]. In addition, CTA is not capable of describing hemodynamics, flow abnormalities or detect micro-embolies. For these reasons, CTA is not routinely used a screening or follow up tool for asymptomatic outpatient cohorts, but rather is differed to when results of screening CDUS are unclear, and to image vascular anatomy prior intervention [9].

Acoustic shadowing (AS) is a common CDUS artifact and is characterized by a signal void behind structures that strongly absorb or reflect ultrasonic waves. A cone of acoustic shadow usually corresponds with fibrocalcific plaques [12]. AS may interfere with the measurement of PSV by obscuring the bifurcation and forcing the PSV measurement distal to the plaque; or by producing an inaccurate PSV reading. This results in reduced sensitivity and specificity and, possibly, loss of reliability. A recent publication has shown that in the presence of AS, CDUS alone appears to be inadequate to accurately determine the degree of carotid stenosis and to identify patients with severe disease, but did not specify the differential effect of AS size on test reliability [13]. Currently, there is no consensus in the literature on how to grade the extent of AS since it is highly variable and dependent on plaque quality, localization and size.

Historically, arterial calcification was considered a marker of advanced atherosclerosis [14] and is associated with age, hypertension, diabetes, smoking, and chronic renal disease [15, 16]. As such, with the increased life expectancy in the general population, a calcified plaque is likely to become much more prevalent which highlights the necessity of understanding its clinical relevance. Current literature considers hyper-echogenic plaques as stable and less likely to cause subsequent ischemic events when compared to hypoechogenic plaque with a rich lipid core [17, 18]. In contrast, there are publications suggesting that an AS serves as marker of atherosclerosis and is predictive of ischemic stroke [16, 19, 20]. Thus, the predictive value of AS and calcified carotid plaques for cardiovascular morbidity remains to be elucidated.

Our objective was to assess the impact of AS artifact and its size on CDUS reliability as compared to CTA. In addition, we sought to identify specific flow patterns distal to the calcified plaque, such as RI and PI that might improve CDUS reliability.

## Methods

This study is a single center retrospective observational study and was approved by the Tel-Aviv Medical Center Ethical Board (IRB).

All patients were identified through the TASMC CDUS unit records registry. The definition of AS artifact was any carotid bifurcation mineralization hindering visualization of the vessel wall.

To be included, patients had to undergo a CDUS exam between Jan-2009 and April 2014. The study’s inclusion criteria were first according to CDUS results- either mention of an AS artifact or high-grade stenosis (defined by PSV > 240 cm/s). These patients were then included in the study cohort only if they had a CTA done within 6 months of the CDUS exam.

Clinical information was retrieved from the study participants’ clinical hospital records. The base line clinical information retrieved for each study participant included – Demographic information, Cardiovascular risk factors (smoking status, diabetes mellitus, dyslipidemia, arterial hypertension), prior cardiovascular morbidity (ischemic heart disease, ischemic stroke or amaurosis fugax), use of antithrombotic medications (Aspirin, Clopidogrel, Warfarin, direct oral anticoagulants), statin use, laboratory data (complete blood count, HBA1C, detailed cholesterol and C reactive protein levels) and Trans Thoracic Echocardiography (TTE) information; when available.

Data on subsequent cardiovascular events and all case mortality rates was collected for an observational period commencing from the CDUS acquisition date and ending in January 1st 2016.

### CDUS image acquisition

All CDUS exams were performed on the same CDUS Phillips device model HD 11, Probe model L12–3 by two certified and experienced technicians. All readings were consistently obtained in an angle adjusted to be parallel with flow direction. All carotid arteries were assessed in 3 planes - frontal, lateral and dorsal positions, using the sternocleidomastoid muscle as an anatomical landmark. In cases of difficulties in visualizing the carotid bifurcation due to sonographic artifacts, specifically an AS, a velocity reading was done distal to the bifurcation in the nearest visualized portion of the internal carotid artery. The CDUS exam data of PSV and EDV readings was retrieved from the computerized CDUS reports including the bifurcation and plaque images taken at the time of the exam.

### CDUS image analysis

**Fig. 1:**
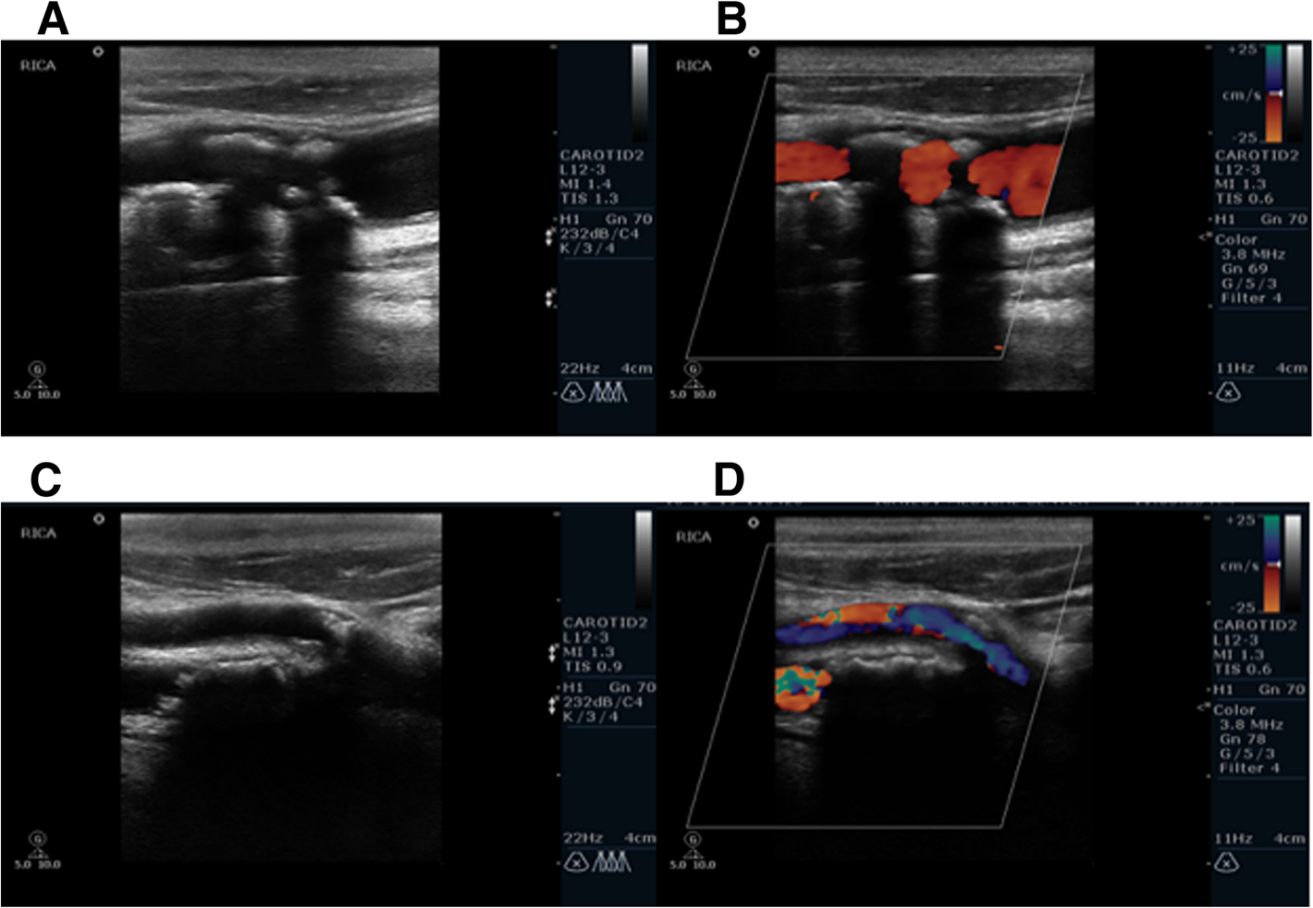
Acoustic Shadow on Sonographic imaging. Legend: Sonographic examples of a small (A + B) and large (C + D) AS (Acoustic shadow) artifact. B-mode ultrasonography of carotid bifurcation demonstrating an atherosclerotic plaque with a small (**a**) and large (**c**) AS artifact. Color Doppler ultrasonography images of carotid artery bifurcation demonstrating an atherosclerotic plaque with a small (**b**) and large (**d**) AS artifact

The CDUS information and images were reviewed by one of the authors (TN), a skilled CDUS technician. The acoustic shadow was graded in a semi-quantitative manner as follows - absent, present, or large. A large shadow was defined as a shadow that covers more than 50% of the vessel diameter when assessing it in a perpendicular angle (Fig. 1) allowing the authors to concentrate on AS obscuring the vessel wall that may influence the CDUS reliability. The reviewer of the CDUS exams was blinded to CTA data and percent stenosis.

Pulsatile Index (PI) was calculated according to the Gosling’s pulsatility index [21].

$$ \mathrm{PI}=\left(\mathrm{PSV}-\mathrm{EDV}\right)/\mathrm{mean}\ \mathrm{flow}\ \mathrm{velocity}. $$

Resistivity Index (RI) was calculated using Pourcelot’s method [22].

$$ \mathrm{RI}=\left(\mathrm{PSV}-\mathrm{EDV}\right)/\mathrm{PSV}. $$

### CTA image acquisition

All CTA exams were obtained with a helical scan technique on the same scanner. Coverage was from arch to vertex, with continuous axial sections parallel to the orbitomeatal line with 0.9 -mm section thickness, 120 kV. Acquisitions were obtained after a single bolus intravenous contrast injection of 80 mL contrast media into an antecubital vein at 3–5 mL/s, auto-triggered by appearance of contrast in a region of interest manually placed in the ascending aorta.

### CTA image analysis

Each CTA exam was reviewed by experienced stroke physicians with extensive knowledge in interpreting CTA scans (ES and HH) that were blinded to the CDUS results. General Electric Centricity® PACS RA1000 Workstation was used for all image analysis. All CTAs were revised and reconstructed to calculate the degree of stenosis. The CTA data was considered to be gold standard as it correlates well with true angiographic percent stenosis on digital subtraction angiography (DSA) [11]. In order to avoid beam-hardening artifact, which can over or under estimate the degree of stenosis, a GE Healthcare system software AW server 2.0 program was used to reconstruct the carotid artery in order to avoid erroneous measurement. For each patient measurement of minimal diameter and surface areas were retrieved from the reconstructed arteries, at fixed anatomical landmarks – distal common carotid (CCA), Bifurcation and plaque, distal extra cranial internal carotid artery. Degree of percent stenosis was calculated using the NASCET trial method [23]:

$$ \mathrm{Percent}\ \mathrm{stenosis}=\left(\mathrm{Distal}\ \mathrm{lumen}\ \mathrm{diameter}-\mathrm{minimal}\ \mathrm{plaque}\ \mathrm{lumen}\ \mathrm{diameter}\right)/\mathrm{distal}\ \mathrm{lumen}\ \mathrm{diameter}. $$

In cases where the distal lumen was collapsed, an arbitrary value of 5 mm was used for the distal diameter, in order to avoid underestimation.

### Statistical analysis

Pearson’s r was used to assess correlations between PSV and degree of stenosis. Bland Altman Plot and reliability analysis was used to test the agreement between measurements of the two diagnostic studies. Arteries with total occlusion were excluded from the analysis. ANOVA test was used for differences in RI and PI between the 3 AS groups. Student’s t-Test and Chi square were used for the univariate analysis of baseline variables and the presence of AS, where appropriate. Sensitivity, specificity, Positive Predictive Value (PPV) and Negative Predictive Value (NPV) were calculated using standard two by two methods, using CTA degree of stenosis as “gold standard”. *P* value less than 0.01 was considered significant. The analysis was done using SPSS version 24 (SPSS Inc., Chicago, IL).

## Results

Out of 15,119 individuals that were scanned in the CDUS unit of the TASMC between January 2009 and April 2014, 967 consecutive patients fulfilled the sonographic inclusion criteria. Six hundred eighty one patients had unilateral severe ICA stenosis regardless of side, out of which 27.7% (*n* = 189) were mentioned to harbor an AS. Sixty eight patients had bilateral severe ICA stenosis, out of which 35.3% (*n* = 24) were mentioned to harbor an AS. Two hundred eighteen patients had an AS artifact with no significant stenosis according to PSV readings.

In order to be included in the study cohort, these patients had to undergo a CTA exam within 6 months of the CDUS exam, which further excluded 683 individuals. 57 (20%) individuals were then excluded due technically inadequate CDUS and/or CTA images containing significant artifacts.

**Fig. 2:**
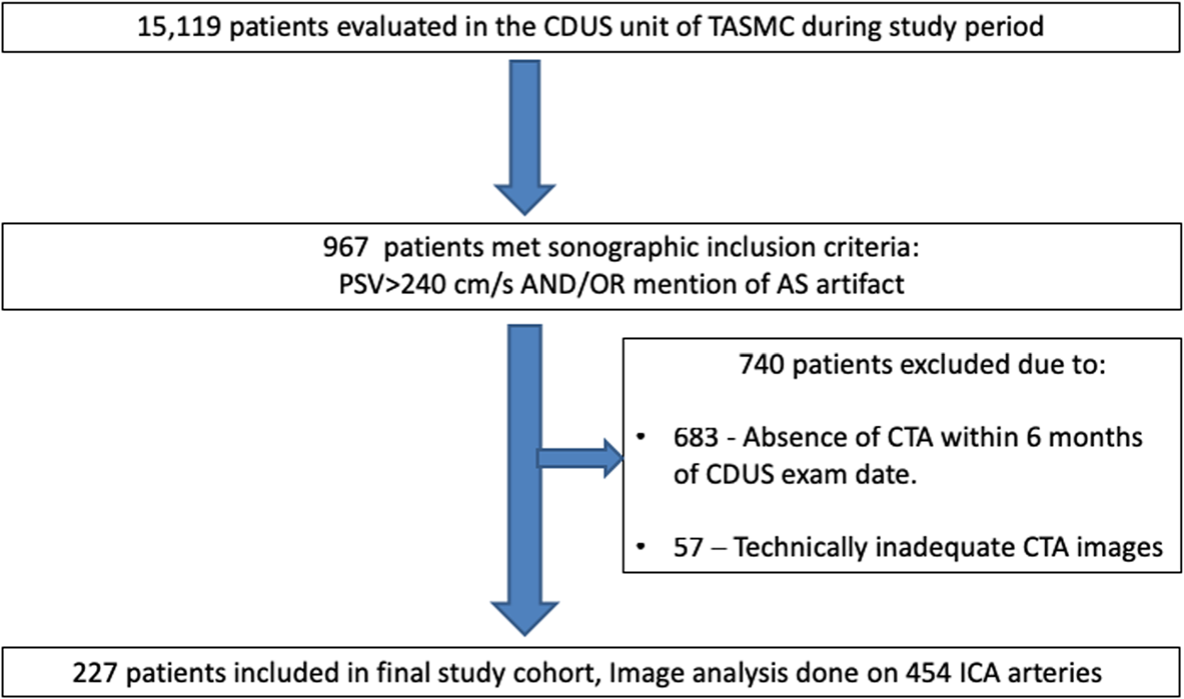
Study Patient selection Flow chart. Legend: CDUS - Color Doppler ultrasound, TASMC -Tel-Aviv Sourasky medical center, AS-Acoustic shadow, PSV- Peak systolic velocity, CTA – CT angiography, ICA-Internal Carotid artery

The final study cohort included 227 patients that met the study’s inclusion criteria; thus, image analysis was carried out on 454 internal carotid arteries. Figure 2 shows patient selection flowchart.

Participants’ base line demographic and clinical characteristics are presented in Table 1. The clinical follow up period for subsequent cardiovascular events and all-cause mortality amounted to 817.83-person years, with a mean follow up period of 43.6 months (SD14.05) after each participant’s CDUS exam date.

**Table 1:** Base line demographic and clinical characteristics of study participants

Age, mean (SD)	68.8 (11.3)
Gender, *n* (%)	
Male	160 (71)
Female	62 (28)
Co morbidities, *n* (%)	
Smoker	Yes - 74 (23.6), Past 46 (20.3)
IHD	111 (49)
HTN	176 (77.5)
AF	26 (11.5)
DM / IGT	91 (40.1)
Stroke / TIA prior to study period	63 (28)
Dyslipidemia	163 (72)
Prior anti thrombotic medications, *n* (%)	
Aspirin	126 (59.8)
Clopidogrel	44 (19.4)
Oral vitamin K antagonists	19 (8.4)
DOAC’S	2 (1)
Statins	133 (59)
Prior symptomatic CS, *n* (%)	100 (44)
Ischemic stroke/TIA	93 (41)
Amurosis Fugax	7 (3)

Study cohort clinical characteristics, *N* = 227 patients included

Legend: *IHD* Ischemic heart disease, *HTN* Arterial hypertension, *AF* Atrial fibrillation, *DM* Diabetes Mellitus, *IGT* Impaired fasting glucose, *TIA* Transient Ischemic attack, *DOAC’S* Direct oral anticoagulants, *CS* Carotid artery stenosis

Table 2 shows the complete arterial imaging data. Nearly half of the arteries (*n* = 195, 43.2%) had an AS artifact present on CDUS imaging, with a large artifact present in 30 arteries (6.7%).

**Table 2:** Carotid arteries clinical and imagining data

Symptomatic CS, *n* (%)	100 (22)
Ischemic stroke/TIA	93 (20.5)
Amurosis Fugax	7 (1.5)
ICA % stenosis on CTA, mean (SD)	35 (30)
ICA stenosis ≥70% on CTA, *n* (%)	62 (15.3)
ICA occlusion per CTA, *n* (%)	49 (10.8)
ICA PSV per CDUS, mean (SD)	206 (154)
ICA shadow per CDUS, *n* (%)	195 (43)
Large	30 (6.6)
ICA PSV ≥240 cm/s per CDUS, *n* (%)	141 (31)

Clinical radiological and sonographic characteristics of Internal Carotid arteries included. This analysis includes 454 arteries from a study cohort of 227 participants

Legend: *CS* Carotid stenosis, *TIA* Transient Ischemic attack, *ICA* Internal carotid, *PSV* Peak systolic velocity, *CTA* CT angiography, *CDUS* Color Doppler ultrasound

Older age was significantly related to the presence of AS artifact (*p* < 0.001). All additional clinical, laboratory and TTE parameters previously detailed, including subsequent cardiovascular events and all-cause mortality rates, did not differ in relation to AS artifact presence and size (data not shown).

**Fig. 3:**
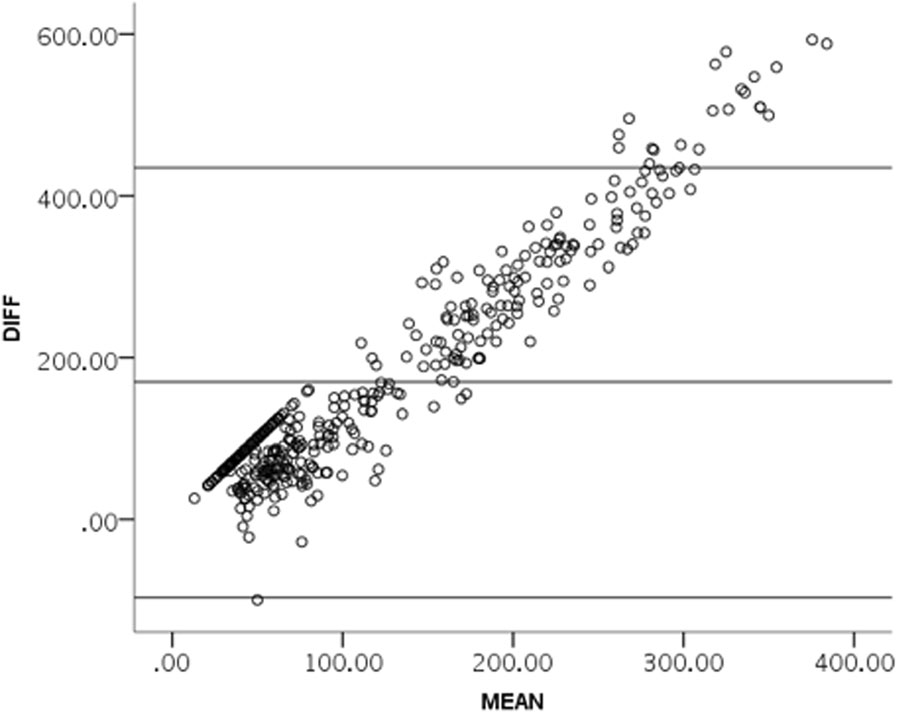
Bland-Altman’s limits of Agreement plot between CT angiography and Color Doppler ultrasonography readings. Bland-Altman’s limits of Agreement plot between CTA and CDUS findings. Legend: SD-standard deviation, DIFF- differences, CDUS – Color Doppler ultrasonography, CTA – CT angiography

For the whole cohort of arteries investigated, our data shows a strong correlation between percent stenosis on CTA and PSV values (Pearson’s r 0.672, *p* < 0.001). Cronbach’s alpha was 0.57 and the Interclass Correlation (ICC) was 0.53 (p < 0.001). The sensitivity and specificity of CDUS PSV values > 240 cm/s in predicting severe carotid stenosis (> 70% stenosis) were 82 and 73% respectively, with a PPV of 35% and NPV of 96%. A Bland-Altman plot of agreement is presented in Fig. 3. The agreement between CDUS and CTA readings decreased with increasing stenosis values in the complete study cohort.

The impact of AS artifact presence and size on CDUS performance is shown in Table 3. As expected, the correlation between CDUS and CTA in arteries without AS was strong and significant (Pearson’s r = 0.41, *p* < 0.001). The presence of a small AS did not affect the correlation or compromise the reliability of the CDUS exam (Pearson’s r = 0.47, *p* <  0.001). On the other hand, a large AS severely affected correlation between CDUS PSV readings and calculated percent stenosis on CTA, with loss of statistical significance (Pearson’s r = 0.24, *p* = 0.27). In addition, the presence of a large AS significantly affected the CDUS reliability; the sensitivity and specificity for detecting severe stenosis dropped to 62%. The reliability analysis likewise showed declining agreement between tests with increased AS artifact intensity.

**Table 3:** Impact of Acoustic Shadow artifact on Color Doppler Ultrasonography reliability

	CTA/CDUS correlations (Pearson’s r)	*P* value	Cronbach’s alpha	ICC	*P* value	CDUS Sensitivity^a^	CDUS Specificity^a^	PPV	NPV
No AS	0.687	< 0.001	0.56	0.515	< 0.001	83.3%	72.8%	33.3%	96.4%
Small AS	0.570	< 0.001	0.61	0.571	< 0.001	89.5%	76.3%	35.4%	98.0%
Large AS	0.49	0.014	0.38	0.382	0.12	62.5%	62.5%	45.5%	76.9%

Correlations between percent stenosis on CDUS and CTA studies according to the presence and extent of the acoustic shadow artifact

Legend: *CDUS* Color Doppler ultrasound, *CTA* CT angiography, *ICC* Inter Class Correlation, *PPV* Positive Predictive Value, *NPV* Negative Predictive Value

^a^ For severe stenosis defined as % stenosis > 70% on CTA, Peak systolic velocity > 240 cm/s

When exploring the ability of other CDUS parameters, such as PI and RI, to improve CDUS accuracy in the presence of an AS, there was no difference in the mean PI and RI between the two groups (*p* = 0.26 and 0.36 respectively). A sub group analysis of arteries with high grade ICA stenosis per CTA (defined as > 70% stenosis) found that in the absence of AS, RI and PI were significantly elevated (*p* = 0.05) a trend that was lost in the presence of AS on CDUS regardless of its size (*p* = 0.86).

## Discussion

In this study we analyzed the correlation between CDUS flow parameters and CTA percent stenosis in patients with carotid artery stenosis and a calcified plaque. CDUS is characterized by high sensitivity and NPV in conjunction with relatively low specificity and PPV [3]. These traits make CDUS an excellent screening tool. In our study cohort CDUS performed as expected and reported in the literature.

The AS artifact represents mineralization of the atherosclerotic plaque resulting in higher tissue density in parts of the plaque. This, potentially hinders accurate flow readings within the stenotic region. A recent publication showed that, in the presence of AS, CDUS alone appears to be inadequate to accurately determine the degree of carotid stenosis, and to identify patients with severe disease [13]. This study had several limitations as stated by its authors –Only 17% of lesions with AS had a concomitant CTA, which could introduce a selection bias. In addition, the gold standard used was CTA cross-sectional area reduction. The amount of flow through a lumen is indeed proportional to its cross-sectional area, but seen as atherosclerotic residual lumens are irregular in shape this method might be less accurate to consider as gold standard and presents further bias [13].

Our data shows that a small amount of mineralization- correlating with a small AS artifact- influences the CDUS wave form and PSV readings only mildly. The overall effect on the diagnostic accuracy is not pronounced- possibly since some Doppler reading is possible within the plaque. This is an important observation, since small AS is very common both in our cohort and in the literature [19].

In contrast, a large AS impacts significantly the reliability of the CDUS exam. We hypothesized that this degradation of the test reliability may be caused by the sonographic block of the bifurcation. In this situation the CDUS technician often needs to assess the flow parameters in a more distal location; that does not necessarily correlate with the real severity of the lumen narrowing within the bifurcation. In these conditions, the exam’s sensitivity and specificity approach the accuracy of a coin toss and may not be relied upon as a screening tool. This supports the conclusion of other publications that the presence of AS reduces the CDUS reliability [13].

Additional flow parameters such as RI and PI may add additional information regarding the flow pattern, and enhance the accuracy of CDUS when assessing a difficult lesion. We found that indeed, RI and PI change substantially in cases with high grade stenosis when AS is absent. In contrast, these flow parameters did not improve CDUS reliability in patients with a large AS and might not be reliable in patients with heavily calcified plaques, although the number of arteries with a large AS was small and this observation might be a result of insufficient power. Furthermore, indirect Stenosis criteria, which might further increase the diagnostic accuracy in the presence of an AS, were not assessed. Still, our data suggests that these parameters cannot be relied upon in patients with heavily-calcified plaques.

From a clinical standpoint, our results show that artery mineralization is probably an age dependent process and degenerative in nature, as attested by the significantly older age of individuals with this artifact. We found no relation to other clinical parameters such as cardiovascular risk factors and morbidity (concomitant hypertension dyslipidemia or diabetes mellitus), laboratory and inflammatory markers or TTE readings. Furthermore, our follow up data failed to show a prognostic value as it did not appear to increase the risk of ipsilateral stroke, subsequent cardiovascular event or all-cause mortality supporting the notion that a calcified plaque is stable and less likely to become symptomatic [17].

Our study does have several limitations. It is a single center study susceptible to selection bias, and the data was retrospectively analyzed. The evaluation of the AS artifact and its magnitude were done in a semi-quantitively fashion and dependent on images taken at the time the study took place. In addition, the number of ICA arteries demonstrating a large AS was relatively small. In addition, we lack CDUS flow readings in intracranial and peri-orbital arteries that was not routinely done in our lab, and therefore cannot add information regarding the degree of stenosis and collateralization in cases of significant CDUS artifacts.

In contrast, our study has some important strengths – The study cohort was derived from a large unselected data base spanning a period of more than five years. All patients underwent CDUS using the same probe by the same experienced technician throughout the study period which minimized inter operator dependent bias. Furthermore, a large proportion of the patients that underwent CDUS had a concomitant CTA as a comparator, in contrast to other published studies, and all CTA images underwent reconstructions to enhance accurate assessment of stenosis. Although there could be a selection bias as part of the retrospective design, it is the largest cohort of patients and ICA arteries to date that discuss the effect of such a common CDUS artifact. We consider this study to be hypothesis generating, and further prospective studies should be done in the future to address the clinical significance and prognostic value of this common artifact.

## Conclusion

The presence of a large AS severely degrades the accuracy and reliability of CDUS. Our data shows that in the presence of a large AS, routine velocity readings may not be suitable as a screening tool for Carotid artery stenosis. In these cases, the physician should refer the patient to a complete and through CDUS exam including doppler-measurement of periorbital arteries and intracranial arteries in addition to other imaging modalities such as CTA or MRA, in order to assess the future stroke risk.

## Data Availability

The datasets used and/or analysed during the current study are available from the corresponding author on reasonable request.

## Abbreviations

AS: Acoustic shadow
CDUS: Color Doppler ultrasound
CS: Carotid stenosis
CTA: CT angiography
DSA: Digital subtraction angiography
EDV: End Diastolic velocity
ICA: Internal carotid artery
NPV: Negative Predictive Value
PI: Pulsatile Index
PPV: Positive Predictive Value
PSV: Peak systolic velocity
RI: Resistivity Index
SD: Standard deviation
TASMC: Tel-Aviv Sorasky medical center
TIA: Transient ischemic attack
TTE: Trans Thoracic Echocardiography
